# Intake and growth histories modulate bone morphology, microarchitecture, and mineralization in juvenile green turtles (*Chelonia mydas*)

**DOI:** 10.1093/conphys/coad080

**Published:** 2023-12-05

**Authors:** Morgan C Abell, José A Sánchez Hernández, Robin Bast, Karen A Bjorndal, Alan B Bolten, Alison M Roark

**Affiliations:** Department of Biology, Furman University, 3300 Poinsett Highway, Greenville, SC 29613 USA; Department of Biology, Hood College, 401 Rosemont Avenue, Frederick, MD 21701 USA; Department of Biology, Hood College, 401 Rosemont Avenue, Frederick, MD 21701 USA; Archie Carr Center for Sea Turtle Research and Department of Biology, University of Florida, PO Box 118525, Gainesville, FL 32611 USA; Archie Carr Center for Sea Turtle Research and Department of Biology, University of Florida, PO Box 118525, Gainesville, FL 32611 USA; Department of Biology, Furman University, 3300 Poinsett Highway, Greenville, SC 29613 USA; Department of Biology, Hood College, 401 Rosemont Avenue, Frederick, MD 21701 USA; Archie Carr Center for Sea Turtle Research and Department of Biology, University of Florida, PO Box 118525, Gainesville, FL 32611 USA

**Keywords:** Compensatory growth, cortical bone, diaphysis, epiphysis, food conversion efficiency, food restriction, realimentation, trabecular bone

## Abstract

Compensatory growth (CG) is accelerated growth that occurs when food availability increases after food restriction. This rapid growth may be associated with sublethal consequences. In this study, we investigated the effects of food restriction and subsequent realimentation and CG on bone structure in juvenile green turtles (*Chelonia mydas*). Turtles were fed *ad libitum* food for 12 weeks (AL), restricted food for 12 weeks (R), or restricted food for 5 weeks followed by *ad libitum* food for 7 weeks (R-AL). R-AL turtles demonstrated partial CG via enhanced food conversion efficiency (FCE) upon realimentation. After the 12th week, gross morphology (GM), microarchitecture, and mineralization of the right humerus of each turtle were analyzed. Many GM measurements (including proximal and maximal bone lengths, bone widths, and shaft thickness), most measurements of bone microarchitecture (excluding cortical and trabecular thickness and trabecular separation), and all mineralization measurements were labile in response to intake. We examined the possibility that changes in nutrient allocation to bone structure during realimentation facilitated CG in previously food-restricted turtles. Restoration of bone lengths was prioritized over restoration of bone widths during CG. Furthermore, restoration of trabecular number, connectivity density, and bone volume fraction was prioritized over restoration of cortical bone volume fraction. Finally, diaphyseal bone mineralization was partially restored, whereas no restoration of epiphyseal bone mineralization occurred during CG. Shifts in nutrient allocation away from certain bone attributes during food restriction that were not rectified when food availability increased probably provided an energy surplus that enhanced the conversion of food to growth and thus powered the CG response. Our study revealed how resource allocation to various bone attributes is prioritized as nutritional conditions change during development. These “priority rules” may have detrimental consequences later in life, indicating that conservation of green turtle foraging grounds should be given high priority.

## Teaser

In juvenile green turtles, nearly all measured bone attributes were labile in response to intake. A shift in nutrient allocation away from bone during food restriction that was not rectified when food availability increased likely provided an energy surplus that enhanced food conversion efficiency and powered a CG response.

## Introduction

Growth rates are largely dictated by resource availability, which can vary across space and time. Because smaller individuals are usually more susceptible to predation and negative outcomes of competition, processes that allow animals to compensate for periods of resource limitation and reduced growth rates may be favored, leading to optimization of individual growth rates (Sohal and Weindruch, 1996). For example, compensatory growth (CG) is a period of accelerated growth in response to restored favorable conditions after a period of unfavorable conditions such as food restriction (Ali *et al.,* 2003). This period of rapid growth decreases the variation in size-at-age among individuals of a species (Atchley, 1984; Bjorndal *et al.,* 2003). Although advantageous in the short term, rapid growth resulting in decreased size disparity among individuals may be associated with changes that are detrimental later in life (Metcalfe and Monaghan, 2001; Mangel and Munch, 2005; Dmitriew, 2011). For example, rapid growth as a juvenile can lead to a higher resting metabolic rate (Criscuolo *et al.,* 2008), increased oxidative stress (Metcalfe and Monaghan, 2003), and thus reduced longevity (Rollo, 2002). CG also has documented consequences for fitness through its negative impacts on mating success and offspring size (Holden *et al.,* 2019).

Although not universal (Kozłowski *et al.,* 2020), CG has been demonstrated in plants, invertebrates, and numerous vertebrates such as fish, reptiles, birds, and mammals (Plavnik and Hurwitz, 1985; Harris *et al.,* 1986; De Block *et al.,* 2007; Tian *et al.,* 2010; Holden *et al.,* 2019; Li *et al.,* 2021). A complete CG response occurs when the size disparity between food-restricted individuals and *ad libitum*-fed individuals is fully rectified upon refeeding (or realimentation), whereas a partial CG response occurs if the size disparity is not fully rectified. The species, developmental stage at which food restriction is imposed, and duration and severity of food restriction can all influence the degree of compensation, leading to differing costs and benefits of rapid growth (Wilson and Osbourn, 1960; Ali *et al.,* 2003; Mitchell, 2007). For example, in Atlantic cod (*Gadus morhua*), the most severe level of food restriction results in the greatest CG response (Jobling *et al.,* 1994). The CG response varies not only in magnitude but also in the mechanisms driving it. Hyperphagia, or increased food consumption, is the most common mechanism of growth compensation (Wang *et al.,* 2000; Nikki *et al.,* 2004), but increased conversion of food to growth (or food conversion efficiency, FCE) during realimentation is also possible (Broekhuizen *et al.,* 1994; Morgan and Metcalfe, 2001; Tian *et al.,* 2010).

CG occurs in aquatic reptiles, although the magnitude of response and the mechanism driving it vary among species. During realimentation, previously food-restricted juvenile Chinese pond turtles (*Chinemys reevesii*) exhibit CG driven by hyperphagia (Wang *et al.,* 2011; Xu *et al.,* 2014). In this species, the CG response is complete when food restriction is moderate and of extended duration (Xu *et al.,* 2014) but only partial after a short period of extreme food restriction (Wang *et al.,* 2011). When protein is specifically restricted, the Chinese softshell turtle (*Pelodiscus sinensis*) undergoes a complete CG response upon realimentation (Xie *et al.,* 2012), but when food in general is restricted, the GC response is only partial (Xie and Niu, 2007).

CG also occurs during the early, oceanic life stage in loggerhead sea turtles (*Caretta caretta*) and green turtles (*Chelonia mydas*). Although the mechanism by which CG occurs in loggerhead sea turtles is unknown (Bjorndal *et al.,* 2003), in green turtles it is driven by enhanced FCE rather than hyperphagia (Roark *et al.,* 2009). For the first few years of life, green turtles occupy oceanic habitats and are omnivorous (Reich *et al.,* 2007). Intake and growth rates (Goshe *et al.,* 2010) during this stage are highly variable because of heterogeneous prey distribution and variation in thermal regimes (Bjorndal *et al.,* 2003). Oceanic-stage turtles also experience nutrient dilution when they ingest marine debris including plastics (McCauley and Bjorndal, 1999; Santos *et al.,* 2020). Periods of restricted intake and thus reduced growth rates, whether resulting from decreased food availability or nutrient dilution, may have substantial fitness effects because body size in green turtles is correlated with predation risk (Salmon and Scholl, 2014) and thus juvenile survival (Chaloupka and Limpus, 2005), as well as both mean and maximal clutch size (Broderick *et al.,* 2003). These fitness impacts are particularly concerning from a conservation standpoint because sea turtle populations are declining throughout the world due to human activities including over-exploitation and habitat loss, with many distinct population segments of green turtles listed as either threatened or endangered (Seminoff *et al.,* 2015).

In addition to documenting the existence of and mechanism underlying CG in juvenile green turtles in a controlled feeding trial, we also previously reported that green turtles that had experienced food restriction and undergone CG restored their body condition with respect to all measured indices (organic matter, nitrogen, lipid, and energy content) except for total body mineral content to match those of turtles fed *ad libitum* throughout the trial (Roark *et al.,* 2009). This lack of restoration of mineral content suggested that soft tissues were prioritized over skeletal tissues and indicated that periods of food restriction may be associated with previously unrecognized sublethal effects in green turtles. Thus, further evaluation of the pattern of recovery of bony tissue is warranted.

In this study, we investigated how intake and growth histories affect bone structure in juvenile green turtles that have experienced food restriction and subsequent CG. We evaluated the gross morphology, microarchitecture, and mineralization of the right humerus from 27 of the juvenile green turtles from our previous CG study (Roark *et al.,* 2009). Our goal was to identify additional sublethal impacts of food restriction and subsequent CG that may have serious conservation implications for this and other vulnerable sea turtle species.

## Materials and Methods

### Feeding trial and sample preparation

**Figure 1:**
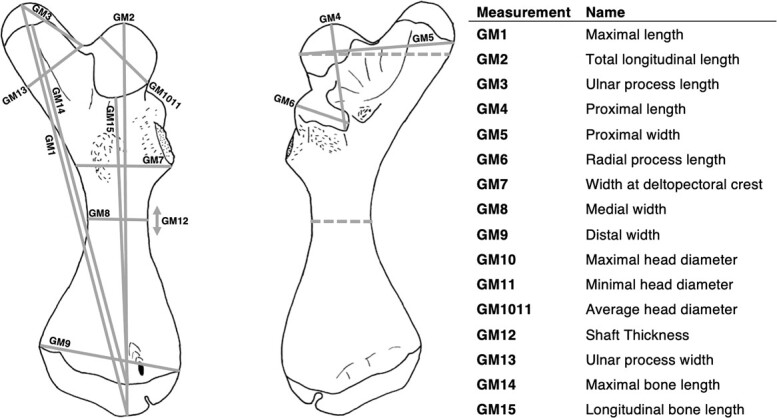
Green turtle (*C. mydas*) humerus, dorsal view (left) and ventral view (right). Solid lines depict gross morphology (GM) measurements with corresponding GM names to the right. Dashed lines on ventral view are the locations of the epiphyseal (top) and diaphyseal (bottom) planes used for μCT analysis and bone histology. Bone measurements and names originally used in Zug *et al.* (1986), with the addition of GM1011, GM14, and GM15. GM1011 is the average of GM10 and GM11 (not shown). Full descriptions of each measurement are in Table S1. Image adapted from Fig. 1 of Zug *et al.* (1986); used with permission.

Samples analyzed in this study were collected from a randomly selected subset of green turtles used in previous research conducted at the Cayman Turtle Centre, Grand Cayman, Cayman Islands (Roark *et al.,* 2009). Briefly, turtles were housed individually and fed daily with turtle pellets (Melick Aquafeed, Catawissa, PA, USA). To establish a daily *ad libitum* intake, all turtles were fed *ad libitum* for seven days before the experimental period. Turtles were then randomly assigned to one of three intake groups (hereafter referred to as treatments): *ad libitum*-fed for 12 weeks (AL, *n* = 7), food-restricted (~50% of *ad libitum* intake) for 12 weeks (R, *n* = 10), and food-restricted for the first  five weeks and then *ad libitum*-fed for the next seven weeks (R-AL, *n* = 10). Minimum straight carapace length (CL; Bolten, 1999) of each turtle was measured weekly; CL ranged from 5.5 to 15.5 cm throughout the trial. After 12 weeks, turtles were euthanized with an overdose ketamine injection, and the right humerus from each turtle (with cartilage in place) was preserved in 70% ethanol. Fifteen gross morphology (GM) measurements were taken to the nearest 0.01 mm in triplicate using digital calipers as described in Zug *et al.* (1986), with the addition of average head diameter (GM1011, the average of GM10 and GM11), GM14, and GM15 (Fig. 1; Supplementary Table S1, available online). Of these 15 measurements, four (GM1, GM2, GM 14, and GM15) were measures of total bone length, three (GM3, GM4, and GM6) were measures of process length, six (GM5, GM7, GM8, GM9, GM1011, and GM13) were measures of bone width, and one (GM12) was a measure of bone thickness. Due to minor damage during dissection, GM3 was estimated for one bone from group R and GM13 for one bone from group AL. Triplicate measurements were averaged for use in statistical analyses.

**Figure 2:**
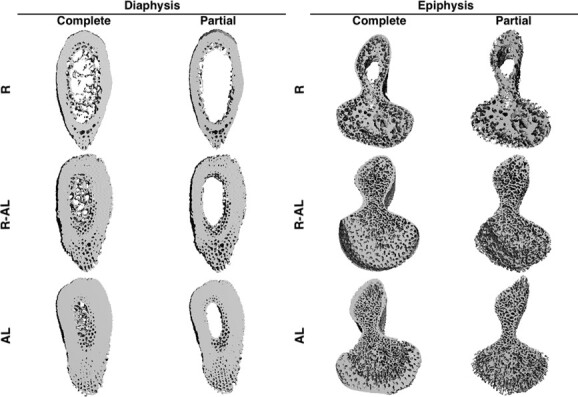
Representative complete and partial micro-computed tomography (μCT) reconstructions of the diaphysis and epiphysis of humeri from juvenile green turtles (*C. mydas*) in three treatment groups: food-restricted (R, *n* = 10); food-restricted then *ad libitum*-fed (R-AL, *n* = 10); *ad libitum*-fed (AL, *n* = 7). Complete sections of diaphysis and epiphysis include both cortical and trabecular bone. Partial diaphyseal sections contain only cortical bone while partial epiphyseal sections include only trabecular bone.

Microarchitecture of cortical and trabecular bone within the diaphysis and epiphysis of each humerus was studied using high-resolution micro-computed tomography (μCT). Three-dimensional reconstructions were generated from X-ray attenuation data captured from multiple viewing angles (Bouxsein *et al.,* 2010; Table 1). Compared to two-dimensional histology techniques, μCT analysis is more objective and useful when assessing bone microarchitecture (Feldkamp *et al.,* 1989; Goulet *et al.,* 1994; Van Rietbergen *et al.,* 1998; Mulder *et al.,* 2004; Chappard *et al.,* 2005). Imaging was conducted at the University of Alabama at Birmingham in the Department of Nutritional Sciences using a μCT40 imaging system (Scanco Medical, Bassersdorf, Switzerland). Complete and partial measurements were reported for both epiphyseal and diaphyseal sections (Fig. 2). Complete measurements included data for both trabecular and cortical bone in both the epiphysis and diaphysis, while partial measurements (generated by manual thresholding) only included data for cortical diaphyseal bone and trabecular epiphyseal bone. The diaphysis of long bones in green turtles contains a thick layer of dense cortical bone surrounding minimal trabecular bone, while the epiphysis consists of mostly trabecular bone surrounded by a thin layer of cortical bone.

**Table 1:** Summary of micro-computed tomography (μCT) measurements used to assess microarchitecture of diaphyseal cortical bone and epiphyseal trabecular bone of juvenile green turtle (*C. mydas*) humeri.

**Measurement**	**Abbreviation**	**Description**	**Units**
**Bone volume density**	BD_d_, BD_e_	Average voxel value of the bone within volume of interest	mg HA/cm^3^
**Bone volume fraction**	BV/TV_d_, BV/TV_e_	Segmented bone volume to the total volume ratio	%
**Trabecular number**	Tb.N	Average number of trabeculae per mm	mm^−1^
**Trabecular thickness**	Tb.Th	Average thickness of trabeculae	mm
**Trabecular separation**	Tb.Sp	Average distance between trabeculae	mm
**Connectivity density**	Conn.D	The degree of connectivity of trabeculae	mm^−3^
**Cortical thickness**	Ct.Th	Average cortical thickness of diaphysis	mm

*Notes:* Descriptions from Bouxsein *et al.* (2010). BD and BV/TV were evaluated in both diaphyseal cortical bone and epiphyseal trabecular bone, indicated by subscripts d and e, respectively. HA, hydroxyapatite.

For each epiphyseal and diaphyseal scan, bone volume fraction of epiphysis or diaphysis (BV/TV_e_ or BV/TV_d_, respectively) was calculated by dividing the partial bone volume (BV) by the complete total volume (TV, including all trabecular and cortical bone). Thus, BV/TV_e_ is the BV of epiphyseal trabecular bone divided by complete epiphyseal TV, while BV/TV_d_ is the BV of diaphyseal cortical bone divided by complete diaphyseal TV.

**Figure 3:**
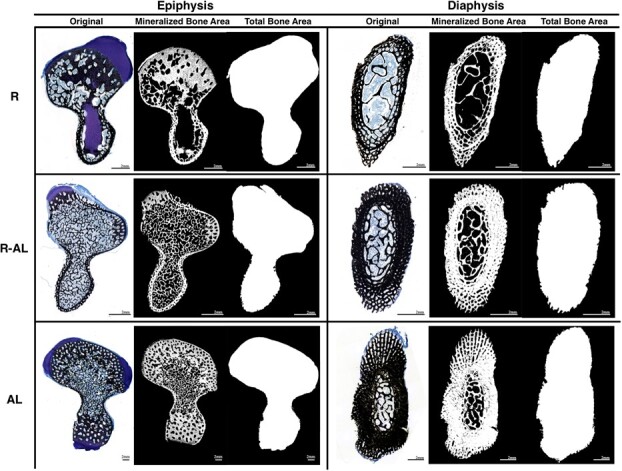
Representative histological samples depicting original images (black = mineralized tissue, blue = unmineralized tissue), images transformed for mineralized bone area, and images transformed for total bone area quantification of the epiphysis and diaphysis of humeri from juvenile green turtles (*C. mydas*) in three treatment groups: food-restricted (R, *n* = 10); food-restricted then *ad libitum*-fed (R-AL, *n* = 10); *ad libitum*-fed (AL, *n* = 7). Scale bars represent 2 mm.

Bones were then sectioned and stained using the Von Kossa staining procedure at Alizée Pathology (Thurmont, MD, USA). Briefly, each bone was embedded in plastic (Technovit 9100) and sectioned through the epiphysis and diaphysis at 5 μm (Fig. 1, dashed lines). Sections were deplasticized, rinsed with distilled water, and soaked in 5% silver nitrate under ultraviolet light for one hour. After being rinsed again in distilled water, slides were placed in 5% sodium thiosulfide for 30 seconds, rinsed with tap water, counterstained with Tetrachrome for six dips, and then rinsed well in tap water. Finally, slides were dehydrated through 95% to 100% ethanol, cleared in xylene, and coverslipped with Cytoseal™. In total, 27 diaphyseal and 27 epiphyseal slides were generated. Slides were photographed under a dissecting microscope alongside a stage micrometer. Using ImageJ software (ImageJ, U.S. National Institutes of Health, Bethesda, Maryland, USA), images were converted to grayscale, and then a threshold was set to exclude cartilage and to select for only mineralized bone pixels. Slides were then made binary, which converted pixels within the threshold (mineralized bone) to white and pixels outside the threshold (cartilage, fluid, and background) to black. The area of white pixels was quantified to determine bone mineralization. To measure total bone cross-sectional area, each section was traced and filled, making the entire area of bone white. Stray white pixels were manually erased. A second threshold was then set to measure and quantify the white pixels. Total bone area (mm^2^) and mineralized area (mm^2^) of digitized slides were determined by two independent observers, and data from both observers were averaged (Fig. 3).

### Statistical analyses

We first used principal component analysis (PCA) with unit variance scaling and singular value decomposition in an attempt to reduce the dimensionality of the GM and μCT data. Principal components with eigenvalues greater than 1.0 were considered significant. Because PCA eigenvectors of all tested variables were weak, especially for GM data, we also used analysis of variance (ANOVA) and analysis of covariance (ANCOVA) or the appropriate nonparametric alternatives to analyze GM and μCT data further and to analyze mineralization.

**Figure 4:**
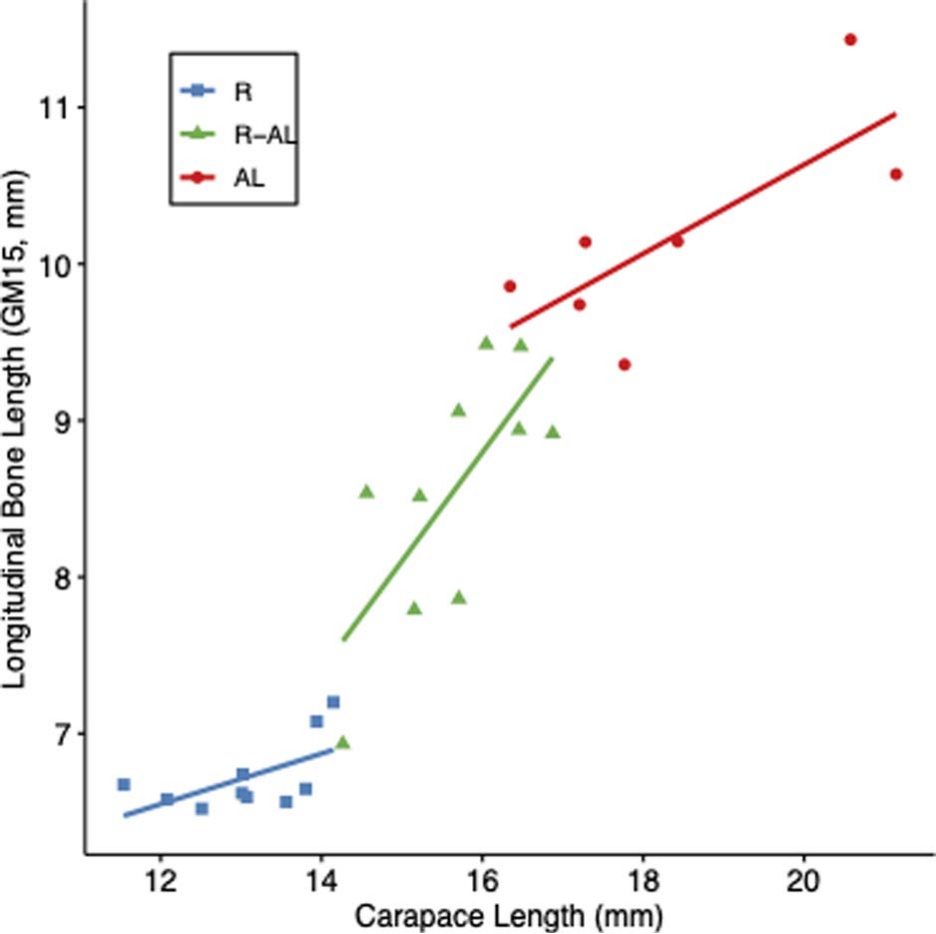
Relationship between longitudinal bone length (GM15) and carapace length (CL) of juvenile green turtles (*C. mydas*) from three treatment groups: food-restricted (R, *n* = 10); food-restricted then *ad libitum*-fed (R-AL, *n* = 10); *ad libitum*-fed (AL, *n* = 7). ANCOVA revealed GM15 did not differ among groups when CL was used as the covariate (*F*_2,23_ = 1.906, *P* = 0.171). Points represent values from individual turtles, and solid lines are lines of best fit for each treatment group (R: R^2^ = 0.49; R-AL: R^2^ = 0.66; AL: R^2^ = 0.85).

Growth rates throughout the feeding trial and thus final body size (CL) of turtles after week 12 differed significantly among treatment groups (Roark *et al.,* 2009); therefore, the effect of treatment on bone gross morphology measurements was analyzed using ANCOVA. Longitudinal bone length (GM15) was used as the covariate in GM ANCOVAs because it did not differ among treatment groups according to ANCOVA when CL was used as a covariate (*F*_2,21_ = 1.906, *P* = 0.171). In other words, for GM15, no significant interaction existed between treatment and body size measured as CL. Moreover, the relationship between CL and GM15 was linear and homogeneous (Fig. 4). While total longitudinal length (GM2) is also a linear, whole bone measurement (Fig. 1), GM15 was chosen as the covariate because it excludes the cartilage at each end of the bone.

Both ANOVA and ANCOVA or the appropriate nonparametric alternatives (a nonparametric Kruskal–Wallis test followed by a Wilcoxon signed-rank post-hoc test and a non-parametric Quade method, respectively) were used to analyze μCT data for differences among groups. ANCOVA or the appropriate nonparametric alternative was used for evaluations of diaphyseal cortical thickness (Ct.Th), with complete diaphyseal TV as the covariate, and for evaluations of epiphyseal trabecular thickness (Tb.Th) and separation (Tb.Sp), with complete epiphyseal TV as the covariate. ANOVA or the appropriate nonparametric alternative was used for evaluation of bone density (BD, reported as average bone volume density in μCT outputs), epiphyseal trabecular number (Tb.N), connectivity density (the degree of trabeculae connectivity; Conn.D), and bone volume fraction (BV/TV; all μCT abbreviations from Bouxsein *et al.,* 2010). Differences in bone mineralization of epiphyseal and diaphyseal bone among groups were compared using ANCOVA or the appropriate nonparametric alternative with total bone cross-sectional area as the covariate.

Data evaluated using ANOVA met the assumptions of normality (Shapiro–Wilk test) and equal variances across treatment groups (Bartlett’s test). Pairwise comparisons were performed using Tukey's honestly significant difference (hsd) post hoc test. When ANOVA assumptions could not be satisfied, a nonparametric Kruskal–Wallis test followed by a Wilcoxon signed-rank post-hoc test was used to compare among groups. Data evaluated using ANCOVA were transformed as necessary to satisfy the assumptions of linearity, homogeneity of regression slopes (i.e., insignificant interaction between treatment and covariate), normality of residuals (Shapiro–Wilk test), and homogeneity of variances (Levene’s test). Pairwise post-hoc comparisons were performed with a Bonferroni multiple comparisons test to identify differences among groups. If ANCOVA assumptions could not be satisfied, a non-parametric Quade method for analysis of covariance was used (www.masungur.com/nancova0.php;Cangür *et al.,* 2018).

XLSTAT (version 2023.1.6) was used for principal component analysis (PCA), including calculation of eigenvalues and eigenvectors, and PCA plots were generated using ClustVis (Metsalu and Vilo, 2015). RStudio version 3.5.2 (R Development Core Team, 2013) was used for all other analyses. For all parametric and nonparametric tests, ⍺ = 0.05. In cases where multiple dependent variables were reported for similar outcomes of interest (the four measures of bone lengths, the three measures of bone process lengths, the six measures of bone widths, and μCT measurements that were reported for both diaphyseal and epiphyseal bone), the cutoff for significance for each omnibus analysis was adjusted by dividing α by the number of dependent variables measured for that outcome of interest.

## Results

**Figure 5:**
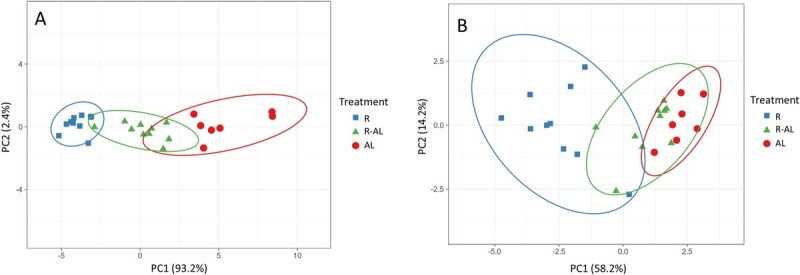
Principal components analysis (PCA) with unit variance scaling and singular value decomposition was used in an attempt to reduce the dimensionality of the gross morphology (A) and μCT (B) data from juvenile green turtles (*C. mydas*) in three treatment groups: food-restricted (R, *n* = 10); food-restricted then *ad libitum*-fed (R-AL, *n* = 10); *ad libitum*-fed (AL, *n* = 7). Principal components 1 (PC1) and 2 (PC2) are shown on the x and y axes, respectively, along with their contributions to total variance. Prediction ellipses indicate that new observations from the same group will fall inside the relevant ellipse with 95% probability. Eigenvectors can be found in Tables S2 and S3.

PCA for gross morphology (GM) data (including all GM variables along with CL) indicated that only the first principal component (PC1) was significant and explained 93.2% (eigenvalue = 15.8) of the variance among individuals. For GM data, all eigenvectors for PC1 were between 0.21 and 0.25 (Table S2); thus, all of the measured response variables correlated comparably but weakly with PC1. For μCT data, the first and second principal components (PC1 and PC2) were significant and explained 58.2% (eigenvalue = 5.24) and 14.2% (eigenvalue = 1.28), respectively, of the variance among individuals. The two variables with eigenvectors whose absolute values were largest for PC1 were trabecular number (eigenvector = 0.423, weak positive correlation with PC1) and trabecular spacing (eigenvector = −0.404, weak negative correlation with PC1) in the epiphysis, and the variable whose absolute value was largest for PC2 was trabecular thickness (eigenvector = 0.662, positive correlation with PC2) in the epiphysis (Table S3). For both GM and μCT data, 95% prediction ellipses for the three treatment groups overlapped but indicated that turtles clustered by treatment (Fig. 5). Due to the lack of strong correlations between response variables and principal components, we were unsuccessful in reducing the dimensionality of the GM and μCT data and thus used analysis of variance (ANOVA) and analysis of covariance (ANCOVA) or the appropriate nonparametric alternatives to analyze these data further.

Five humerus GM measurements were not significantly affected by treatment when analyzed using ANCOVA with GM15 as a covariate. Ulnar process length (GM3, Quade: *F*_2,23_ = 1.407, *P* = 0.264), radial process length (GM6, ANCOVA: *F*_2,23_ = 0.812, *P* = 0.456), and medial width (GM8, Quade: *F*_2,23_ = 0.356, *P* = 0.704) did not differ among groups and thus were not significantly affected by treatment. Maximal length (GM1) and total longitudinal length (GM2), both of which include cartilage, also did not differ among groups (ANCOVA: *F*_2,23_ = 0.798, *P* = 0.462 and Quade: *F*_2,23_ = 0.644, *P* = 0.534, respectively).

**Figure 6:**
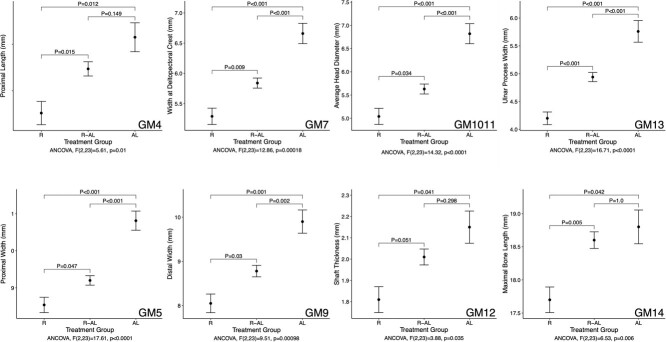
Gross morphology (GM) measurements (millimetres), expressed as estimated marginal means ± SE, of juvenile green turtles (*C. mydas*) that differed significantly among three treatment groups: food-restricted (R, *n* = 10); food-restricted then *ad libitum*-fed (R-AL, *n* = 10); *ad libitum*-fed (AL, *n* = 7). All measurements were evaluated using ANCOVA with GM15 as the covariate and post hoc comparisons were performed with a Bonferroni *P*-value adjustment for multiple comparisons using the emmeans package in R. GM13 and GM14 were log-transformed to satisfy the assumptions of ANCOVA and estimated marginal means and standard errors were then back-transformed for presentation. Refer to Fig. 1 for measurement descriptions.

Other humerus gross morphology (GM) measurements were significantly affected by treatment when analyzed using ANCOVA with GM15 as a covariate (Fig. 6). Proximal length (GM4) and maximal bone length (GM14) were significantly shorter in R turtles than in both R-AL and AL turtles (ANCOVA: *F*_2,23_ = 5.61, *P* = 0.01 and ANCOVA: *F*_2,23_ = 6.53, *P* = 0.006, respectively). Proximal width (GM5, ANCOVA: *F*_2,23_ = 17.61, *P* < 0.0001), width at the deltopectoral crest (GM7, ANCOVA: *F*_2,23_ = 12.86, *P* = 0.0002), distal width (GM9, ANCOVA: *F*_2,23_ = 9.51, *P* = 0.001), average head diameter (GM1011, ANCOVA: *F*_2,23_ = 14.32, *P* < 0.0001), and ulnar process width (GM13, ANCOVA: *F*_2,23_ = 16.71, *P* < 0.0001) also differed significantly among treatment groups. These measurements were all longest in AL turtles, shortest in R turtles, and intermediate in R-AL turtles. Shaft thickness (GM12) was significantly less in R turtles than in AL turtles (ANCOVA: *F*_2,23_ = 3.88, *P* = 0.035).

**Figure 7:**
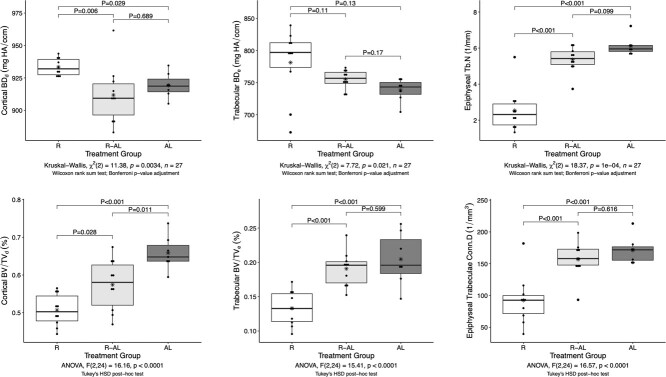
Micro-computed tomography (μCT) measurements that were significantly affected by treatment. Treatment groups are as follows: food-restricted (R, *n* = 10); food-restricted then *ad libitum*-fed (R-AL, *n* = 10); *ad libitum*-fed (AL, *n* = 7). Each dot represents a data point from an individual turtle. Boxplots indicate median (bold line within box), interquartile range (height of the box), minimum and maximum values within 1.5 times the interquartile range beyond the box (vertical whiskers), and arithmetic mean (asterisk). Refer to Supplementary Table S4 (available online) for measurements that were not significantly affected by treatment. HA, hydroxyapatite.

Of the microarchitecture measurements (Fig. 7), diaphyseal bone volume fraction (BV/TV_d_, ANOVA: *F*_2,24_ = 16.16, *P* < 0.0001) was the only one that differed significantly among all three groups; it was highest in AL turtles, intermediate in R-AL turtles, and lowest in R turtles. In contrast, epiphyseal bone volume fraction (BV/TV_e_, ANOVA: *F*_2,24_ = 15.41, *P* < 0.0001) was significantly lower in R turtles than in AL and R-AL turtles. Diaphyseal cortical bone volume density (BD_d_, Kruskal–Wallis test: χ^2^ = 11.38, *P* = 0.0034) was significantly higher in R turtles than in AL and R-AL turtles, whereas epiphyseal trabecular bone volume density (BD_e_) did not differ among groups according to post hoc analysis despite a *P*-value for the omnibus Kruskal Wallis test (χ^2^ = 7.72) of 0.021. Epiphyseal trabecular connectivity density (Conn.D, ANOVA: *F*_2,24_ = 16.57, *P* < 0.0001) and epiphyseal trabecular number (Tb.N, Kruskal–Wallis test: χ^2^ = 18.37, *P* = 1 × 10^−4^) were also significantly lower in R turtles than in R-AL and AL turtles. Both epiphyseal trabecular thickness (Tb.Th, Quade: *F*_2,21_ = 0.304, *P* = 0.741) and trabecular separation (Tb.Sp, Quade: *F*_2,21_ = 0.626, *P* = 0.543) did not differ significantly among groups. Additionally, cortical thickness of the diaphysis (Ct.Th, ANCOVA: *F*_2,21_ = 2.71, *P* = 0.088) did not differ significantly among groups.

**Figure 8:**
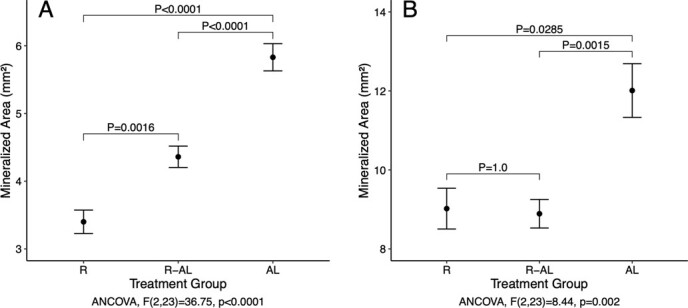
Mineralized area (square millimetres) of humeri from juvenile green turtles (*C. mydas*) in each group in both the diaphysis (A) and the epiphysis (B). Mineralized area is presented as estimated marginal means ± SE. Treatment groups are as follows: food-restricted for 12 weeks (R, *n* = 10); food-restricted then *ad libitum*-fed (R-AL, *n* = 10); *ad libitum*-fed (AL, *n* = 7). Omnibus *P*-values reflect results of ANCOVA using total bone area as the covariate. Pairwise *P*-values were adjusted using Bonferroni’s correction for multiple comparisons.

Bone mineralization differed significantly among groups in both the epiphysis and the diaphysis (Fig. 8). Mineralization in the epiphysis of AL turtles was significantly greater than mineralization in the epiphysis of both R-AL and R turtles (ANCOVA: *F*_2,21_ = 8.44, *P* = 0.002). However, diaphyseal bone mineralization was greatest in AL turtles, intermediate in R-AL turtles, and lowest in R turtles (ANCOVA: *F*_2,21_ = 36.75, *P* < 0.001).

## Discussion

Oceanic-stage sea turtles face periods of limited food intake due to stochastic food availability (Bjorndal *et al.,* 2003). When food becomes readily available after a period of food shortage, many species of turtles undergo CG (Bjorndal *et al.,* 2003; Xie and Niu, 2007; Roark *et al.,* 2009; Xu *et al.,* 2014). Accelerated growth during the period of realimentation may be advantageous because survival often increases with increasing body size (Blanckenhorn, 2000), but it may also be associated with long-term sublethal consequences (Marcil-Ferland *et al.,* 2013).

In a previous study (Roark *et al.,* 2009), we demonstrated that CG in R-AL turtles did not eliminate the body size disparity with AL turtles. However, R-AL turtles were able to partially restore body size and to completely restore body condition with respect to all measured indices (organic matter, nitrogen, lipid, and energy content) except for total body mineral content, which was significantly lower in R-AL turtles than in AL turtles. The mechanism underlying this period of CG was enhanced FCE (Roark *et al.,* 2009).

The enhanced FCE that R-AL turtles demonstrated during the period of CG could have resulted from lower investment in bone growth, mineralization, or density during realimentation. Sublethal but long-term costs may result from decreased allocation of energy to growth and maintenance of tissues such as bone. The effects of differences in intake and growth can be age-dependent and regionally specific among various bone structures, sections, and types (Starck and Chinsamy, 2002; Bize *et al.,* 2006; Sheng *et al.,* 2007). In the current study, we found that changes in nutrient allocation facilitated growth compensation in previously food-restricted juvenile green turtles. We also determined which attributes of bone are labile in response to intake and growth histories and which are fixed. Our findings reveal how resource allocation to various bone attributes is prioritized as nutritional conditions change during development.

The lack of an effect of treatment on maximal length (GM1), total longitudinal length (GM2), and longitudinal bone length (GM15) demonstrates that maintenance of overall bone length is generally prioritized regardless of treatment. However, maximal bone length (GM14) and proximal length (GM4) were smaller in R turtles than in AL and R-AL turtles. GM4 and GM14 include the bony portions of the radial and ulnar processes, respectively, and these processes are apparently more labile than bone length in response to treatment. During realimentation, allocating nutrients from the increased food intake to lengthen these processes is prioritized, allowing R-AL turtles to achieve size-adjusted maximal bone lengths and proximal lengths that were comparable to those in AL turtles and longer than those in R turtles by the end of the study. Thus, a period of accelerated growth did not occur at the cost of decreased bone length. Apparently, maintenance of a consistent ratio of humerus length to CL is a priority to allow greater swimming speeds, efficiency, and predator avoidance.

Like humerus length (GM4 and GM14), humerus thickness (GM12) did not differ between R-AL and AL turtles, indicating that humerus thickness is also prioritized during rapid growth. Prioritization of thickness meets the critical need for humerus strength for the power strokes of the long foreflippers of green turtles. Compared to humerus lengths, humerus widths and measurements of the bone processes were more labile in response to treatment. Various width measurements at each end of the humerus were reduced in response to food restriction and only somewhat rectified during realimentation. These measurements included proximal width (GM5), width at the deltopectoral crest (GM7), distal width (GM9), average head diameter (GM1011), and ulnar process width (GM13). Therefore, maintenance of bone length and thickness are prioritized over maintenance of bone width during periods of nutritional stress, but treatment-induced deficits in bone widths are at least partially rectified when intake and growth increase.

Bone volume fraction (BV/TV) was labile in response to treatment in both the diaphysis (cortical BV/TV_d_) and the epiphysis (trabecular BV/TV_e_), and deficits in bone volume fraction induced by food restriction were rectified completely in the epiphysis but only partially in the diaphysis upon realimentation. BD of cortical bone in the diaphysis, but not of trabecular bone in the epiphysis, differed among groups. Specifically, slow growth was associated with increased cortical BD and fast growth was associated with decreased density, which suggests a constant rate of cortical bone deposition regardless of growth rate. This relationship deserves further investigation.

Analysis of epiphyseal trabecular thickness, number, separation, and connectivity density revealed that only trabecular number and connectivity density were affected by treatment and were highly prioritized during CG. This study focused on humeri, but trabecular bone is present in other locations. Trabecular bone is sandwiched between layers of cortical bone in the carapace, which is critical for protection in turtles. The energy from compressive deformation to the carapace is absorbed by this trabecular bone (Ampaw *et al.,* 2019). Restoration of trabecular bone once food levels are returned to normal would be advantageous because this type of bone dictates the stress-bearing capacity of the carapace (Fratzl *et al.,* 2016).

Relative to *ad libitum* feeding, food restriction was associated with decreased mineralization in both the epiphysis and the diaphysis. These deficits were partially rectified during realimentation in the diaphysis but not the epiphysis, indicating that mineral allocation to the bone shaft, where strength is critical for power strokes, is prioritized over mineral allocation to the ends of the bone. Decreased investment in bone mineralization during and after a period of food restriction could improve efficiency of converting food to growth; however, this shift in allocation could have lasting negative consequences on bone strength.

Bone mineralization and μCT measurements revealed differences in the bone attributes that are prioritized during periods of food restriction and subsequent CG in the epiphysis and the diaphysis. For example, BD was maintained in the trabecular bone of the epiphysis regardless of treatment, whereas BD of the cortical bone in the diaphysis was dictated by growth rate. In contrast, bone volume fraction (or the percentage of a bone region occupied by bone rather than marrow or other tissue types) decreased after a period of food restriction and was restored completely in the epiphysis but only partially in the diaphysis. Although restoration of BV/TV was greatest in the epiphysis, the degree of mineralized bone restoration during CG was greater in the diaphysis than in the epiphysis. Mineral investment may be decreased in other bony structures, such as skull, ribs, plastron, or carapace. Mineralization is especially important in the ribs of turtles because they serve as a cushion against high strain forces (Achrai and Wagner, 2013). Weakening of body structures caused by CG is not unique to turtles; for example, sunfish (*Lepomis gibbosus*) experience decreased scale strength as a cost of rapid growth (Arendt *et al.,* 2001).

Among all bone structure measurements, humerus lengths (GM1, GM2, GM3, GM6, and GM15), medial width (GM8), diaphyseal cortical thickness (Ct.Th), and some attributes of the epiphysis (BD_e_, Tb.Th, and Tb.Sp) were not affected by treatment, indicating a consistent pattern of nutrient allocation to maintenance of humerus length and some epiphyseal features regardless of intake and growth rates. Other measurements, including total length (GM14), proximal length (GM4), BD_d_, BV/TV_e_, epiphyseal Tb.N, and epiphyseal Conn.D, were sensitive to a period of food restriction but were corrected during CG to the extent that differences between R-AL and AL turtles were not significant by the end of the study. Taken together, these results indicate that lengthening of the long bones and strengthening of the epiphysis of these bones (critical for maintaining the essential joint with the scapula) seem to be prioritized as nutritional conditions fluctuate. On the contrary, width measurements at the ends of the bone (GM5, GM7, GM9, GM1011, and GM13), shaft thickness (GM12), diaphyseal cortical bone volume fraction (BV/TV_d_), and diaphyseal bone mineralization were only partially rectified during the realimentation period. Of the bone attributes we studied, only one (mineralized area of the epiphysis) was diminished by food restriction and not at all rectified during realimentation. A shift in nutrient allocation away from certain bone attributes during food restriction, combined with a failure to reprioritize these attributes when food availability increased, could have driven the enhanced FCE demonstrated by turtles as they underwent CG and the lower total mineral content of R turtles at the end of the trial.

This study revealed the effects of intake and growth rates on bone structure and the extent to which the effects of food restriction can be remediated by subsequent refeeding in juvenile green turtles. The shifts we documented in nutrient allocation and resulting effects in bone structures may be associated with long-term costs that could not be evaluated in a 12-week study. Further research into the long-term costs of CG, especially with respect to bone structure and in animals like turtles that demonstrate indeterminate growth, is needed. Deficits incurred from poor diet during the juvenile stage due to habitat loss or ingestion of marine debris could have lasting consequences, suggesting that conservation of green turtle foraging grounds should be given high priority.

## Supplementary Material

Web_Material_coad080
