# Supplementary material for: Intake and growth histories modulate bone morphology, microarchitecture, and mineralization in juvenile green turtles (Chelonia mydas)
Source: Conserv Physiol. 2023 Dec 5;11(1):coad080. doi: 10.1093/conphys/coad080 (PMC10699740; doi:10.1093/conphys/coad080)
Supplement: Web_Material_coad080 [file web_material_coad080.zip › abell et al cons phy supp material v4.pdf]

SUPPLEMENTARY MATERIAL

Supplementary Table 1. Summary of gross morphology (GM) measurements of the right humeri of juvenile green turtles (*Chelonia mydas*). Bones were measured in triplicate with calipers.
Notes: Bone measurements and names originally used in Zug *et al*. (1986), with the addition of GM1011, GM14, and GM15. GM1011 is the average of GM10 and GM11.

Measurement	Name	Description
**GM1**	Maximal length	Distance from proximal tip of ulnar process to distal articular cartilage surface
**GM2**	Total longitudinal length	Distance from proximal cartilage surface of head to distal articular surface of cartilage (parallel to longitudinal axis)
**GM3**	Ulnar process length	Distance from proximal tip of ulnar process to juncture of head and process
**GM4**	Proximal length	Distance from proximal surface of head to distal edge of radial process (parallel to longitudinal axis)
**GM5**	Proximal width	Distance from preaxial surface of head to postaxial surface of ulnar process (perpendicular to longitudinal axis)
**GM6**	Radial process length	Distance from pre- to postaxial edges of radial process, diagonal to longitudinal axis
**GM7**	Width at deltopectoral crest	Transverse distance of shaft from pre- to postaxial surfaces at deltopectoral crest
**GM8**	Medial width	Transverse distance from pre- to postaxial surfaces at point of minimum width
**GM9**	Distal width	Transverse distance from pre- to postaxial surfaces at juncture of articular condyles with shaft
**GM10**	Maximal head diameter	Maximal diameter of head
**GM11**	Minimum head diameter	Minimal diameter of head
**GM1011**	Average head diameter	Average diameter of head
**GM12**	Shaft Thickness	Minimal depth in middle of shaft, in vicinity of medial width, perpendicular to longitudinal axis
**GM13**	Ulnar process width	Distance from postaxial surface of ulnar process to juncture of head and process, diagonal to longitudinal axis
**GM14**	Maximal bone length	Distance from bone at proximal tip of ulnar process to distal articular bone surface
**GM15**	Longitudinal bone length	Distance from proximal surface of bone at the head to distal articular surface of bone

Supplementary Table 2. Eigenvectors for principal component 1 (PC1) for gross morphology (GM) measurements of the right humeri and carapace length (CL) of 27 juvenile green turtles (*Chelonia mydas*). PC1 was the only significant component and explained 93.2% (eigenvalue = 15.8) of the variance among individuals. Refer to Figure 1 for measurement descriptions and Figure 5 for the PCA plot.

**Measurement**	**PC1**
GM1	0.244
GM2	0.248
GM3	0.210
GM4	0.245
GM5	0.249
GM6	0.224
GM7	0.246
GM8	0.244
GM9	0.248
GM10	0.244
GM11	0.245
GM1011	0.248
GM12	0.240
GM13a	0.248
GM14	0.245
GM15	0.244
CL	0.249

Supplementary Table 3. Eigenvectors for principal components 1 (PC1) and 2 (PC2) for µCT data for the right humeri of 27 juvenile green turtles (*Chelonia mydas*). PC1 and PC2 were the only significant components and explained 58.2% (eigenvalue = 5.24) and 14.2% (eigenvalue = 1.28), respectively, of the variance among individuals. Refer to Table 1 for measurement descriptions and Figure 5 for the PCA plot.

**Measurement**	PC1	PC2
	**Diaphysis**	
BD	-0.234	-0.382
BV/TV	0.302	0.284
Ct.Th	0.312	0.136
	**Epiphysis**	
BD	-0.264	-0.385
BV/TV	0.369	0.185
Tb.n	0.423	-0.126
Tb.Th	-0.238	0.662
Tb.Sp	-0.404	0.131
Conn.D	0.390	-0.318

Supplementary Table 4. Results of micro-computed tomography (μCT) measurements expressed as mean (SE) of epiphyseal and diaphyseal bone in the humerus of juvenile green turtles from three treatment groups: food-restricted (R, n=10); food-restricted followed by ad libitum-fed (R-AL, n=10); ad libitum-fed (AL, n=7). Refer to Table 1 for measurement descriptions.

	**Diaphysis**			
**Measurement**	Omnibus P	AL	R-AL	R
BD	**3.38e-3**	804.4^{A} (9.708)	911.8^{A} (22.346)	933.4^{B} (6.578)
BV/TV	**3.59e-5**	0.659^{A} (0.020)	0.573^{B} (0.027)	0.507^{C} (0.027)
Ct.Th†	0.088	NA	NA	NA
	**Epiphysis**			
**Measurement**	Omnibus P	AL	R-AL	R
BD	**0.021**	738.4^{A} (18.146)	755.1^{A} (14.938)	781.3^{A} (53.934)
BV/TV	**4.95e-5**	0.205^{A} (0.01)	0.191^{A} (0.014)	0.133^{B} (0.014)
Conn.D	**3.02e-5**	171.19^{A} (11.72)	156.69^{A} (15.28)	92.49^{B} (15.28)
Tb.N	**1.03e-4**	6.107^{A} (0.528)	5.355^{A} (0.717)	2.540^{B} (1.194)
Tb.Th*	0.741	NA	NA	NA
Tb.Sp*	0.543	NA	NA	NA

Notes: Within each row, significant omnibus p-values are bolded, and values with different superscript letters are significantly different according to ANOVA or the non-parametric equivalent with a Bonferroni p-value adjustment for multiple comparisons. While the epiphyseal bone density omnibus p-value was significant, no significant differences were found among groups. NA = not applicable because omnibus p-value was greater than 0.05.

† ANCOVA was used with complete diaphyseal TV as covariate; results are expressed as estimated marginal means.

* The non-parametric Quade method was used with complete epiphyseal TV as covariate; results are expressed as estimated marginal means.
